# Supplementary material for: Androgen receptor (AR) antagonism triggers acute succinate‐mediated adaptive responses to reactivate AR signaling
Source: EMBO Mol Med. 2021 Mar 11;13(5):e13427. doi: 10.15252/emmm.202013427 (PMC8103094; doi:10.15252/emmm.202013427)
Supplement: Supplementary file 1 — Appendix [file EMMM-13-e13427-s004.pdf]

APPENDIX

Androgen Receptor (AR) antagonism triggers succinate-mediated adaptive response to reactivate AR

Neetu Saxena[1], Eliana Beraldi[1], Ladan Fazli[1], Syam Prakash Somasekharan[1], Hans Adomat[1], Fan Zhang[1], Chidi Molokwu[1], Anna Gleave[2], Lucia Nappi[2], Kimberly Nguyen[1], Pavn Brar[1], Nicholas Nikesitch[1], Yuzhuo Wang[1,2], Colin Collins[1,2], Poul H Sorensen[2], and Martin Gleave*[1,2].

1. *Vancouver Prostate Centre, Vancouver, BC, Canada.*
2. *Department of Urologic Sciences, University of British Columbia, Vancouver, BC, Canada*

Correspondence to:
Email: m.gleave@ubc.ca

Table of contents:

Appendix Table No.	Description
S1	List of reagents
S2	List of siRNAs
S3	List of antibodies
S4	List of probes for qRT-PCR
S5	List of probes for ChIP
S6	List of probes for EMSA
S7	List of statistical method used and p values

Appendix Table S1: List of reagents used in this study.

**Reagents**	**Company**	**Catalogue Number**
SB 203580	Sigma-Aldrich	S8307
Genistein	Sigma-Aldrich	G6649
Dimethyl succinate	Sigma-Aldrich	W239607
Dimethyl malonate	Sigma-Aldrich	136441
Dorsomorphin	Sigma-Aldrich	P5499
ENZA	Haoyuan chemexpress co. Ltd.	HY-70002
STO-609	Santa Cruz Biotech	CAS 52029-86-4
Darolutamide (ODM-201)	Orion Corporation and Bayer, Berlin, Germany	
Cycloheximide	Calbiochem	239765
IVM	Aikon International Ltd.	
VPC-14449	Calbiochem	532996

Appendix Table S2: List of siRNAs used in this study.

Si RNA	Company and catalogue no.	Sequence for custom designed siRNAs
Scr	Thermo Scientific Dharmacon<br>Custom designed	5′ CAGCGCUGACAACAGUUUCAU3′
SDHA	Ambion<br>4392420, Id: s12656	Commercial (sequences not provided by the vendor)
SDHB	Ambion<br>4392420, Id: s224589	Commercial (sequences not provided by the vendor)
HIF1α	Ambion<br>4390824, Id: s6541	Commercial (sequences not provided by the vendor)
Hsp27	Thermo Scientific Dharmacon<br>oligo id: BERED-000083	5′ GCUGCAAAAUCCGAUGAGACdTdT3′
AR	Thermo Scientific Dharmacon<br>oligo id: CTM-345 BERED-000101	5′ UCAAGGAACUCGAUCGUAUUU3′
AMPKα1/2	Santacruz Biotech<br>sc-45312	Commercial (sequences not provided by the vendor)

Appendix Table S3: Antibodies used in this study.

Antibodies	Company	Catalogue Number	Application and dilution
PSA	Cell Signaling	5365	Western (1: 500)
p-AMPKα	Cell Signaling	2535	Western (1:1000), TMA (1:100)
T-AMPKα	Cell Signaling	2603	Western (1:500)
P-p38	Cell Signaling	4511	Western (1: 1000), TMA (1:100)
T-p38	Cell Signaling	9212	Western (1:1000)
Vinculin	Sigma-Aldrich	V4505	Western (1:500)
SDHA	Abcam	Ab14715	Western (1:5000)
SDHB	Abcam	Ab14714	Western (1:5000)
T-CaMKK2	Abcam	Ab124096	Western (1:1000)
AR	Santa Cruz	Sc-7305	Western (1:1000)
Actin	EMD Millipore	MAB1501R	Western (1:5000)
T-Hsp27	Enzo Life Sciences	ADI-SPA-803F	Western (1:5000)
p-Hsp27	Enzo Life Sciences	ADI-SPA-523	Western (1:1000)
SDHA	Novus	NBP32594	TMA (1:200)
SDHB	Novus	NB600-793	TMA (1:400)
p-Hsp27	Cell Signaling	2401	TMA (1:25)
T-Hsp27	Stressgen	ADI-SPA-803-F	TMA (1:6000)
PSA	Santa Cruz	sc-7638	TMA (1:50)
Synaptophysin	Abcam	ab32127	TMA (1:500)
AR	Santa Cruz	sc-816	TMA (1:50)
CD56	CellMarque	156R-94	TMA (1:200)
CgA	Epitomics	AC-0037	TMA (1:100)
p-CaMKK2	Cell Signaling	12818S	TMA (1:100)

Appendix Table S4: List of Taqman probes used for qRT-PCR.

**Gene**	**TaqMan probes (Catalogue number)**
SDHA	Hs00417200_ml
SDHB	Hs0104282_ml
AR	Hs00171172_ml
FKBP5	Hs01561006_m1
KLK3 (PSA)	Hs00426859_g1
NKX3-1	Hs00171834_m1
GAPDH	Hs02758991_g1
Vin	Hs00419715_m1
ACTB	Hs99999903_m1

Appendix Table S5: List of qRT-PCR primers for ChIP.

**Gene**	**qRT-PCR primers for ChIP**
SDHA_F	5′ CATCTGGAGCAGGAGCTGTC 3′
SDHA_R	5′ TTGCAGACGAGCAGATTCCG 3′
SDHB_F	5′ AGCCTTCTCTGACTCTTCACTCA 3′
SDHB_R	5′ TCAGACATGGTATTAGGGGCTAGT 3′
GAPDH_F	5′ TCTTTTGCGTCGCCAGCCGAG 3′
GAPDH_R	5′ TGACCAGGCGCCCAATACGAC 3′
PSA_F	5′ TGCAGTTGGTGAGTGGTCAT 3′
PSA_R	5′ ATGGAGAAAGTGGCTGTTGC 3′

Appendix Table S6: List of probes used for EMSA.

**Gene**	**Probes for EMSA**
SDHA_F	5′/5IRD700/GCGACTT**GTGC**TGGCTGAGGGA 3′
SDHA_R	5′/5IRD700/TCCCTCAGCCA**GCAC**AAGTCGC3′
SDHA_F (mutant)	5′/5IRD700/GCGACTT**ACAT**TGGCTGAGGGA3′
SDHA_R (mutant)	5′/5IRD700/TCCCTCAGCCA**ATGT**AAGTCGC3′
SDHB_F	5′/5IRD700/AGAGCTCCCT**GTAC**TTTCCCAACA3′
SDHB_R	5′/5IRD700/TGTTGGGAAA**GTAC**AGGGAGCTCT3′
SDHB_F (mutant)	5′/5IRD700/AGAGCTCCCT**ACGT**TTTCCCAACA3′
SDHB_R (mutant)	5′/5IRD700/TGTTGGGAAA**ACGT**AGGGAGCTCT3′

*The bold nucleotides in the primers were highlighted by genomatrix analysis as key binding nucleotides in AREs with AR and been replaced by alternative purine/pyrimidine nucleotides in the mutated primers.

Appendix Table S7: List of statistical method used and p values.

Figure number	P value	Method	Correction
**Figure 1**			
Figure 1C			
LNCaP: CSS vs. Ctrl	0.0035	One-way ANOVA	Tukey correction
LNCaP: ENZA vs. Ctrl	<0.0001	One-way ANOVA	Tukey correction
LAPC4: CSS vs. Ctrl	0.0005	One-way ANOVA	Tukey correction
LAPC4: ENZA vs. Ctrl	<0.0001	One-way ANOVA	Tukey correction
Figure 1D			
LNCaP: CSS vs. Ctrl	0.0045	One-way ANOVA	Tukey correction
LNCaP: ENZA vs. Ctrl	0.0095	One-way ANOVA	Tukey correction
LAPC4: CSS vs. Ctrl	0.0064	One-way ANOVA	Tukey correction
LAPC4: ENZA vs. Ctrl	0.0003	One-way ANOVA	Tukey correction
Figure 1E ENZA			
SDHA: 6h vs. 0h	0.0593	One-way ANOVA	Tukey correction
SDHA: 12h vs. 0h	0.0187	One-way ANOVA	Tukey correction
SDHA: 24h vs. 0h	0.0127	One-way ANOVA	Tukey correction
SDHB: 6h vs. 0h	0.0274	One-way ANOVA	Tukey correction
SDHB: 12h vs. 0h	0.0037	One-way ANOVA	Tukey correction
SDHB: 24h vs. 0h	0.0055	One-way ANOVA	Tukey correction
PSA: 6h vs. 0h	0.1523	One-way ANOVA	Tukey correction
PSA: 12h vs. 0h	<0.0001	One-way ANOVA	Tukey correction
PSA: 24h vs. 0h	<0.0001	One-way ANOVA	Tukey correction
Figure 1E R1881			
SDHA: 6h vs. 0h	0.0147	One-way ANOVA	Tukey correction
SDHA: 12h vs. 0h	0.0038	One-way ANOVA	Tukey correction
SDHA: 24h vs. 0h	<0.0001	One-way ANOVA	Tukey correction

SDHB: 6h vs. 0h	0.0056	One-way ANOVA	Tukey correction
SDHB: 12h vs. 0h	0.0301	One-way ANOVA	Tukey correction
SDHB: 24h vs. 0h	0.0014	One-way ANOVA	Tukey correction
PSA: 6h vs. 0h	0.011	One-way ANOVA	Tukey correction
PSA: 12h vs. 0h	0.0032	One-way ANOVA	Tukey correction
PSA: 24h vs. 0h	0.0072	One-way ANOVA	Tukey correction
Figure 1F			
PSA: R1881 AR vs. CSS AR	0.0070	One-way ANOVA	Tukey correction
SDHA: R1881 AR vs. CSS AR	<0.0001	One-way ANOVA	Tukey correction
SDHB: R1881 AR vs. CSS AR	0.0177	One-way ANOVA	Tukey correction
Figure 1G			
SDHA-M vs. SDHA	0.0003	Two tailed Unpaired student's T-Test	
SDHB-M vs. SDHB	0.001	Two tailed Unpaired student's T-Test	
**Figure 2**			
Figure 2C			
siSDHA vs. siScr	0.0078	One-way ANOVA	Tukey correction
siSDHB vs. siScr	0.0100	One-way ANOVA	Tukey correction
siSDHA+ENZA vs. siScr+ENZA	<0.0001	One-way ANOVA	Tukey correction
siSDHB+ENZA vs. siScr+ENZA	0.0092	One-way ANOVA	Tukey correction
Figure 2D			
SDHA: siSDHA vs. siScr	<0.0001	Two tailed Unpaired student's T-Test	
SDHB: siSDHB vs. siScr	0.0003	Two tailed Unpaired student's T-Test	
PSA: siSDHA vs. siScr	0.001	Two tailed Unpaired student's T-Test	
PSA: siSDHB vs. siScr	0.043	Two tailed Unpaired student's T-Test	

FKBP5: siSDHA vs. siScr	0.001	Two tailed Unpaired student's T-Test	
FKBP5: siSDHB vs. siScr	0.001	Two tailed Unpaired student's T-Test	
NKX3.1: siSDHA vs. siScr	0.045	Two tailed Unpaired student's T-Test	
NKX3.1: siSDHB vs. siScr	0.012	Two tailed Unpaired student's T-Test	
Figure 2F			
SDHA vs. Empty	<0.0001	One-way ANOVA	Tukey correction
SDHB vs. Empty	<0.0001	One-way ANOVA	Tukey correction
SDHA+ENZA vs. Empty+ENZA	<0.0001	One-way ANOVA	Tukey correction
SDHB+ENZA vs. Empty+ENZA	<0.0001	One-way ANOVA	Tukey correction
Figure 2G			
siSDHA vs. siScr	0.0002	One-way ANOVA	Tukey correction
siSDHB vs. siScr	<0.0001	One-way ANOVA	Tukey correction
siSDHA+ENZA vs. siScr+ENZA	0.0013	One-way ANOVA	Tukey correction
siSDHB+ENZA vs. siScr+ENZA	<0.0001	One-way ANOVA	Tukey correction
Figure 2H			
SDHA+ENZA vs. Empty+ENZA	<0.0001	One-way ANOVA	Tukey correction
SDHB+ENZA vs. Empty+ENZA	<0.0001	One-way ANOVA	Tukey correction
**Figure 3**			
Figure 3C			
siHsp27 vs. siSDHA	0.0194	One-way ANOVA	Tukey correction
siSDHA+ENZA vs. siScr+ENZA	0.0406	One-way ANOVA	Tukey correction
siHsp27+ENZA vs. siSDHA+ENZA	0.0002	One-way ANOVA	Tukey correction
siSDHA+siHsp27+ENZA vs. siHsp27+ENZA	0.0025	One-way ANOVA	Tukey correction
Figure 3E			

AR: siScr+SB+ENZA vs. siScr+ENZA	0.0226	One-way ANOVA	Tukey correction
AR: siSDHB+Gen+ENZA vs. siScr+ENZA	0.0456	One-way ANOVA	Tukey correction
Figure 5C			
AR: NHT treated vs. untreated	0.0007	One-way ANOVA	Tukey correction
AR: CRPC vs. untreated	<0.0001	One-way ANOVA	Tukey correction
SDHA: NHT treated vs. untreated	<0.0001	One-way ANOVA	Tukey correction
SDHA: CRPC vs. untreated	<0.0001	One-way ANOVA	Tukey correction
p-CaMKK2: NHT treated vs. untreated	0.0017	One-way ANOVA	Tukey correction
p-CaMKK2: CRPC vs. untreated	0.0006	One-way ANOVA	Tukey correction
p-AMPK: NHT treated vs. untreated	0.0002	One-way ANOVA	Tukey correction
p-AMPK: CRPC vs. untreated	<0.0001	One-way ANOVA	Tukey correction
p-p38: CRPC vs. untreated	<0.0001	One-way ANOVA	Tukey correction
p-Hsp27: NHT treated vs. untreated	<0.0001	One-way ANOVA	Tukey correction
p-Hsp27: CRPC vs. untreated	<0.0001	One-way ANOVA	Tukey correction
**Figure 6**			
Figure 6A			
Acute CX vs. Pre-CX	0.0006	One-way ANOVA	Tukey correction
CRPC vs. Acute CX	0.0001	One-way ANOVA	Tukey correction
Figure 6B			
2.5 µM IVM+ENZA vs. ENZA	<0.0001	One-way ANOVA	Tukey correction
5 µM IVM+ENZA vs. ENZA	<0.0001	One-way ANOVA	Tukey correction
Figure 6C			
5 µM IVM vs. Ctrl	0.0001	One-way ANOVA	Tukey correction
2.5 µM IVM+ENZA vs. ENZA	0.0079	One-way ANOVA	Tukey correction
5 µM IVM+ENZA vs. ENZA	0.0008	One-way ANOVA	Tukey correction
Figure 6D			

siSDHA+IVM vs. siSDHA	0.006	One-way ANOVA	Tukey correction
siScr+IVM+ENZA vs. siScr+ENZA	0.0235	One-way ANOVA	Tukey correction
siScr+IVM vs. siScr	0.0011	One-way ANOVA	Tukey correction
siSDHA vs. siScr+IVM	0.0013	One-way ANOVA	Tukey correction
siScr+IVM+ENZA vs. siSDHA+ENZA	0.0002	One-way ANOVA	Tukey correction
Figure 6E			
PSA: IVM vs. CX	<0.0001	One-way ANOVA	Tukey correction
Tumor volume: IVM vs. CX	<0.0001	One-way ANOVA	Tukey correction
**Figure EV-1**			
Figure EV1-E			
AR silencing			
SDHA: siAR vs. siScr	0.0002	Two tailed Unpaired student's T-Test	
SDHB: siAR vs. siScr	0.0005	Two tailed Unpaired student's T-Test	
AR: siAR vs. siScr	0.0071	Two tailed Unpaired student's T-Test	
CSS			
SDHA: 3days vs. 0days	0.0005	One-way ANOVA	Tukey correction
SDHA: 5days vs. 0 days	0.0003	One-way ANOVA	Tukey correction
SDHB: 3days vs. 0days	<0.0001	One-way ANOVA	Tukey correction
SDHB: 5days vs. 0days	<0.0001	One-way ANOVA	Tukey correction
PSA: 3days vs. 0days	<0.0001	One-way ANOVA	Tukey correction
PSA: 5days vs. 0days	<0.0001	One-way ANOVA	Tukey correction
ODM-201			
SDHA: 6h vs. 0h	0.0027	One-way ANOVA	Tukey correction
SDHA: 12h vs. 0h	0.0009	One-way ANOVA	Tukey correction
SDHA: 24h vs. 0h	0.0002	One-way ANOVA	Tukey correction

SDHB: 6h vs. 0h	0.0473	One-way ANOVA	Tukey correction
SDHB: 12h vs. 0h	0.0228	One-way ANOVA	Tukey correction
SDHB: 24h vs. 0h	0.0113	One-way ANOVA	Tukey correction
PSA: 6h vs. 0h	0.0001	One-way ANOVA	Tukey correction
PSA: 12h vs. 0h	<0.0001	One-way ANOVA	Tukey correction
PSA: 24h vs. 0h	<0.0001	One-way ANOVA	Tukey correction
**Figure EV2**			
Figure EV2-A			
siSDHA vs. siScr	<0.0001	One-way ANOVA	Tukey correction
siSDHB vs. siScr	<0.0001	One-way ANOVA	Tukey correction
Figure EV2-B			
SDHA vs. empty	0.0018	One-way ANOVA	Tukey correction
SDHB vs. empty	0.0007	One-way ANOVA	Tukey correction
Figure EV2-C			
siScr+ENZA vs. siScr	0.0020	One-way ANOVA	Tukey correction
siSDHA+ENZA vs. siScr+ENZA	0.0001	One-way ANOVA	Tukey correction
siSDHB+ENZA vs. siScr+ENZA	0.0011	One-way ANOVA	Tukey correction
Figure EV2-D			
Empty+ENZA vs. empty	<0.0001	One-way ANOVA	Tukey correction
SDHB+ENZA vs. empty+ENZA	<0.0001	One-way ANOVA	Tukey correction
Figure EV2-E			
siSDHA vs. siScr	0.0076	One-way ANOVA	Tukey correction
siSDHB vs. siScr	0.0048	One-way ANOVA	Tukey correction
Figure EV2-G			
DMM vs. Ctrl	<0.0001	One-way ANOVA	Tukey correction
DMM+ENZA vs. Ctrl+ENZA	0.0143	One-way ANOVA	Tukey correction
**Figure EV3**			

Figure EV3-B			
SDHB: siSDHB+ENZA vs. siScr+ENZA	0.005	One-way ANOVA	Tukey correction
SDHB: siSDHB+siHIF1α+ENZA vs. siScr+ENZA	0.006	One-way ANOVA	Tukey correction
HIF1α: siSDHB+siHIF1α+ENZA vs. siScr+ENZA	<0.0001	One-way ANOVA	Tukey correction
Figure EV3-D			
siSDHA+siHsp27+ENZA vs. siSDHA+ENZA	0.0004	One-way ANOVA	Tukey correction
siSDHB+siHsp27+ENZA vs. siSDHA+ENZA	<0.0001	One-way ANOVA	Tukey correction
Figure EV3-F			
V16D vs. LNCaP	0.0001	One-way ANOVA	Tukey correction
MR49F vs. LNCaP	0.0033	One-way ANOVA	Tukey correction
**Figure EV4**			
Figure EV4-A			
siSDHA+ENZA vs. siScr+ENZA	0.0057	One-way ANOVA	Tukey correction
siSDHB+ENZA vs. siScr+ENZA	0.0227	One-way ANOVA	Tukey correction
SDHA+ENZA vs. siScr+ENZA	0.0031	One-way ANOVA	Tukey correction
SDHB+ENZA vs. siScr+ENZA	<0.0001	One-way ANOVA	Tukey correction
Figure EV4-B			
siSDHA+ENZA vs. siScr+ENZA	<0.0001	One-way ANOVA	Tukey correction
siSDHB+ENZA vs. siScr+ENZA	0.0091	One-way ANOVA	Tukey correction
Figure EV4-D			
siSDHA+siAMPK+ENZA vs. siSDHA+ENZA	<0.0001	One-way ANOVA	Tukey correction
**Figure EV5**			
Figure EV5-D			
AR: CRPC vs. castration-sensitive	0.0222	One-way ANOVA	Tukey correction

AR: NEPC vs. castration-sensitive	<0.0001	One-way ANOVA	Tukey correction
p-CaMMK2: CRPC vs. castration-sensitive	0.0113	One-way ANOVA	Tukey correction
p-Hsp27: NEPC vs. castration-sensitive	0.0068	One-way ANOVA	Tukey correction
