# Supplementary material for: Androgen receptor (AR) antagonism triggers acute succinate‐mediated adaptive responses to reactivate AR signaling
Source: EMBO Mol Med. 2021 Mar 11;13(5):e13427. doi: 10.15252/emmm.202013427 (PMC8103094; doi:10.15252/emmm.202013427)
Supplement: Supplementary file 2 — Expanded View Figures PDF [file EMMM-13-e13427-s006.pdf]

Expanded View Figures

Figure EV1. ARPI inhibits expression of SDH catalytic subunits SDHA and SDHB.

A LNCaP cells were treated with 10 μM ENZA for the indicated time points and proteomic analysis was performed using Tandem mass tagging assay. ENZA reduced expression of various TCA cycle enzymes including different SDH subunits (SDHA, SDHB, SDHC, and SDHD) with simultaneous increase in the expression of different glycolytic enzymes.

B AR downregulation by either siRNA (upper panel) or antisense oligonucleotides (lower panel) in LNCaP or LAPC4 cells decreased SDHA and SDHB subunit protein expression.

C Analysis of SDHB RNA expression from cBioPortal database shows deletions as a major genetic aberration. Total samples (n): 1324, missense mutations: 5, deep deletions: 9, shallow deletions: 115, and gain: 8.

D cBioPortal analysis shows genomic alteration frequency in *SDHA* (upper left panel) and *SDHB* genes (upper right panel) as well as mutations detected in SDHA (bottom left panel) and SDHB (bottom right panel) proteins in prostate adenocarcinoma.

E RT–PCR analysis showing decreased transcript levels of *SDHA* and *SDHB* genes in LNCaP cells after AR silencing (left panel) or other ARPI (CSS; right panel, and 10 μM ODM-201; bottom panel).

F ARE sequences as detected by Genomatrix Promoter analysis in the promoter region of *SDHA*, *SDHB*, *SDHD*, *SDHAF2*, and *HSCB* genes. Hexamer half-sites have been indicated in uppercase letters.

Data information: ENZA: enzalutamide, CSS: charcoal stripped serum, ODM-201 Darolutamide. Data shown as means ± SD of three independent exepriments. Statistical analysis was performed using two tailed unpaired Student's *t*-test (AR silencing) or one-way ANOVA followed by Tukey's test (CSS, ODM-201). *$P < 0,05$, **$P < 0.01$ compared between groups. Exact *P* values are reported in the Appendix Table S7.

 © 2021 The Authors

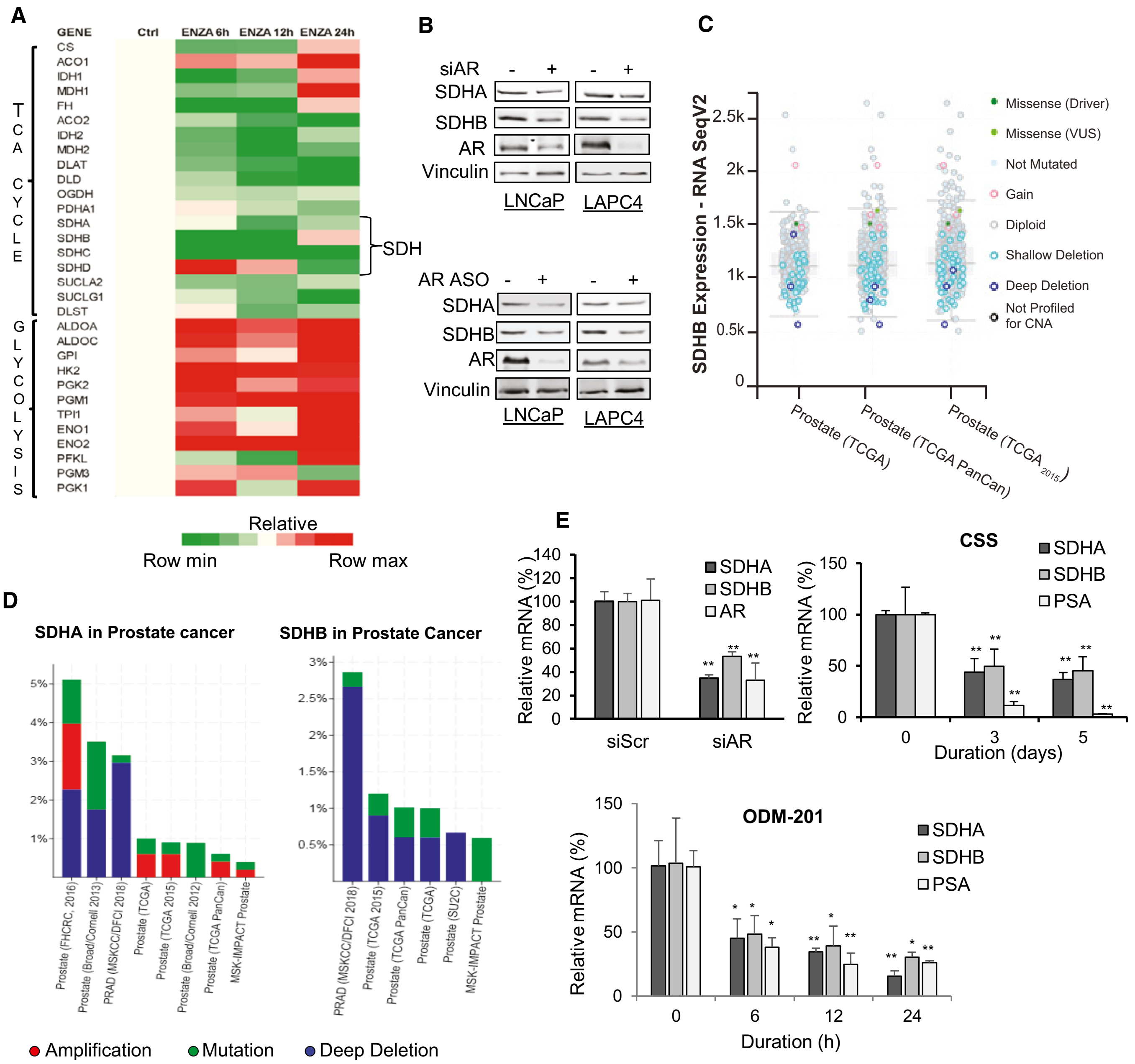

SDHA Protein Change	Mutation Type	Mutation Status
E240Q	Missense	Somatic
I235F	Missense	Somatic
R329*	Nonsense	NA
R329*	Nonsense	Somatic
R329*	Nonsense	.
G242R	Missense	NA
S214N	Missense	NA
S214N	Missense	Somatic
S214N	Missense	.
H296P	Missense	NA
H296P	Missense	NA
G276S	Missense	Somatic
V425G	Missense	Somatic
P332T	Missense	Somatic

SDHB Protein Change	Mutation Type	Mutation Status
S100F	Missense	Somatic
S100F	Missense	Somatic
S100F	Missense	Somatic
S100F	Missense	NA
W47*	Nonsense	NA
R230C	Missense	Somatic
R230C	Missense	.
T250I	Missense	.
T250I	Missense	Somatic
T250I	Missense	Somatic

F

Genomatrix Promoter analysis

Gene	ARE Sequence
SDHA	caagagcgactTGTGCTgg
SDHB	ccagagctcccTGTACTtt
SDHC	Not detected
SDHD	cagggacttggTATTCTtt
SDHAF1	Not detected
SDHAF2	ttggacctcagTCTTCTca
HSCB	gcagaagaaccTGTCCTcc

Figure EV1.

Figure EV2. Inhibition of SDH activity leads to intracellular succinate accumulation and increased AR protein and activity.

A, B SDHA and SDHB silencing increases (A), while subunit overexpression decreases (B) intracellular succinate levels in LNCaP cells.
C, D SDHA and SDHB silencing in the presence of ENZA increased succinate levels more than ENZA alone (C). Conversely, overexpression of these subunits inhibited ENZA-mediated accumulation of succinate in LNCaP cells (D).
E SDHA and SDHB subunit silencing in 22Rv1 cells increased AR-V7 activity measured by UBE2C luciferase assay.
F Inhibition of SDH activity in LNCaP cells treated with different DMM concentrations for 24 h increased AR protein levels in presence and absence of ENZA.
G Treatment with 2 mM DMM partially rescued AR activity after ENZA in LNCaP cells.
H Overexpression of SDHA decreases AR protein levels in LAPC4 (left panel) and 22Rv1 (right panel) cells.

Data information: ENZA: enzalutamide, DMM: Dimethyl malonate. Data shown as mean ± SD of three independent experiments. Statistical analysis was performed using one-way ANOVA followed by Tukey's test. **P* < 0.05 and ***P* < 0.01 compared between groups. Exact *P* values are reported in the Appendix Table S7.

© 2021 The Authors

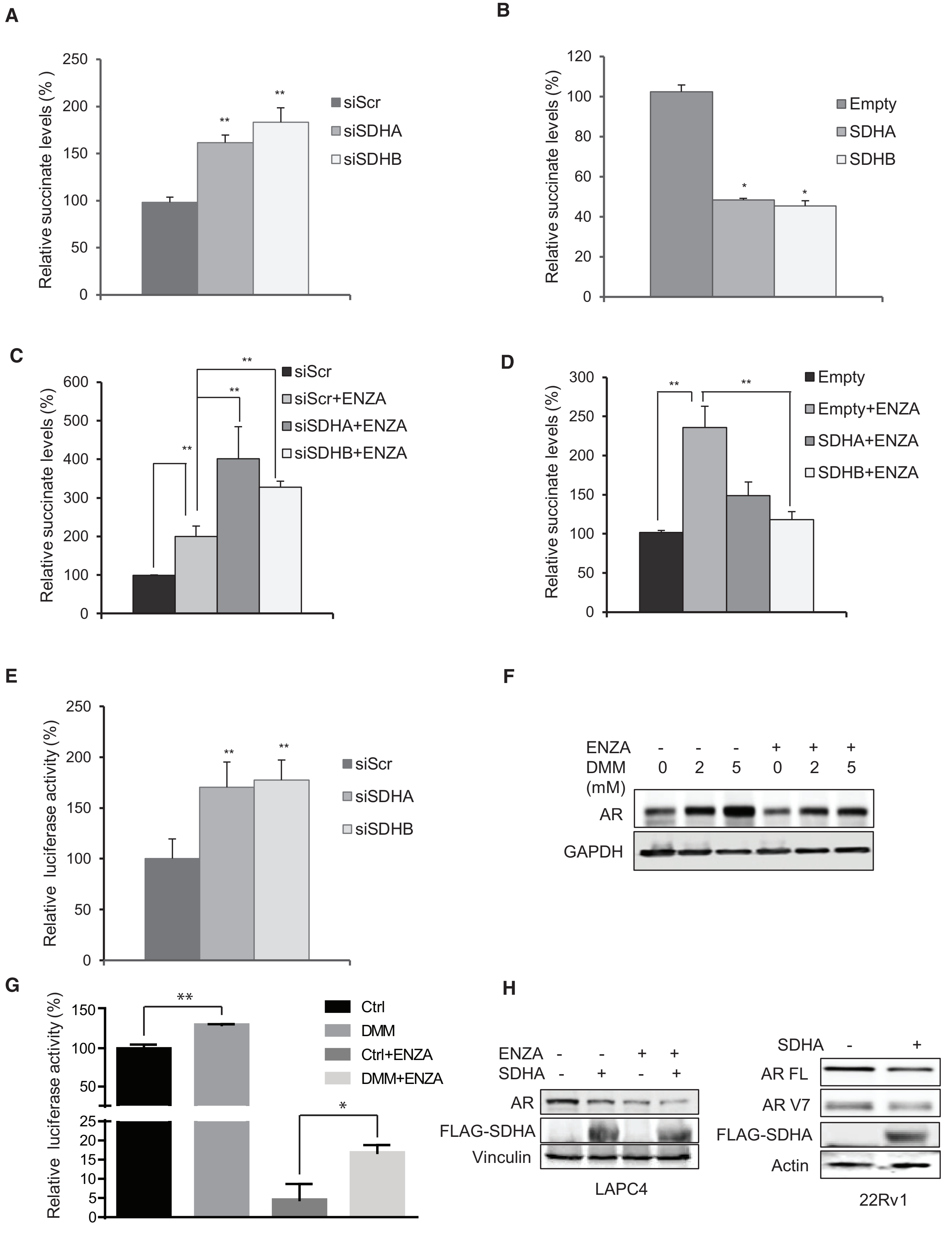
A
Relative succinate levels (%)
250
200
150
100
50
0
siScr
siSDHA
siSDHB
**
**
B
Relative succinate levels (%)
120
100
80
60
40
20
0
Empty
SDHA
SDHB
*
*
C
Relative succinate levels (%)
600
500
400
300
200
100
0
siScr
siScr+ENZA
siSDHA+ENZA
siSDHB+ENZA
**
**
**
D
Relative succinate levels (%)
300
250
200
150
100
50
0
Empty
Empty+ENZA
SDHA+ENZA
SDHB+ENZA
**
**
E
Relative luciferase activity (%)
250
200
150
100
50
0
siScr
siSDHA
siSDHB
**
**
F
ENZA - - - + + +
DMM 0 2 5 0 2 5
(mM)
AR
GAPDH
G
Relative luciferase activity (%)
150
100
50
25
20
15
10
5
0
Ctrl
DMM
Ctrl+ENZA
DMM+ENZA
**
*
H
ENZA - - + +
SDHA - + - +
AR
FLAG-SDHA
Vinculin
LAPC4
SDHA - +
AR FL
AR V7
FLAG-SDHA
Actin
22Rv1

Figure EV2.

Figure EV3. Hsp27 contributes to increased AR levels and cell viability post SDH repression.

A Effect of SDHB silencing on AR protein levels is more pronounced in LNCaP grown in hypoxia (1% O_2) vs normoxia (21% O_2).
B Co-silencing of HIF1α and SDHB does not reduce AR levels significantly at mRNA (left panel) or protein levels (right panel) in LNCaP cells.
C ENZA treatment for 12 h increases p-p38 and p-Hsp27 levels in LNCaP cells.
D Hsp27 co-silencing reverses cell survival benefits imparted by SDHA/SDHB silencing under ENZA stress.
E Overexpression of SDHB subunit in LNCaP cells reduces p-p38/p-Hsp27 axis as well as AR protein levels.
F V16D and MR49F cells maintain higher SDH activity compared to LNCaP cells in androgen deprived condition.

Data information: ENZA: enzalutamide Data shown as mean ± SD of three independent experiments. Statistical analysis was performed using one-way ANOVA followed by Tukey's test. **P* < 0.05 and ***P* < 0.01 compared between groups. Exact *P* values are reported in the Appendix Table S7.

 © 2021 The Authors

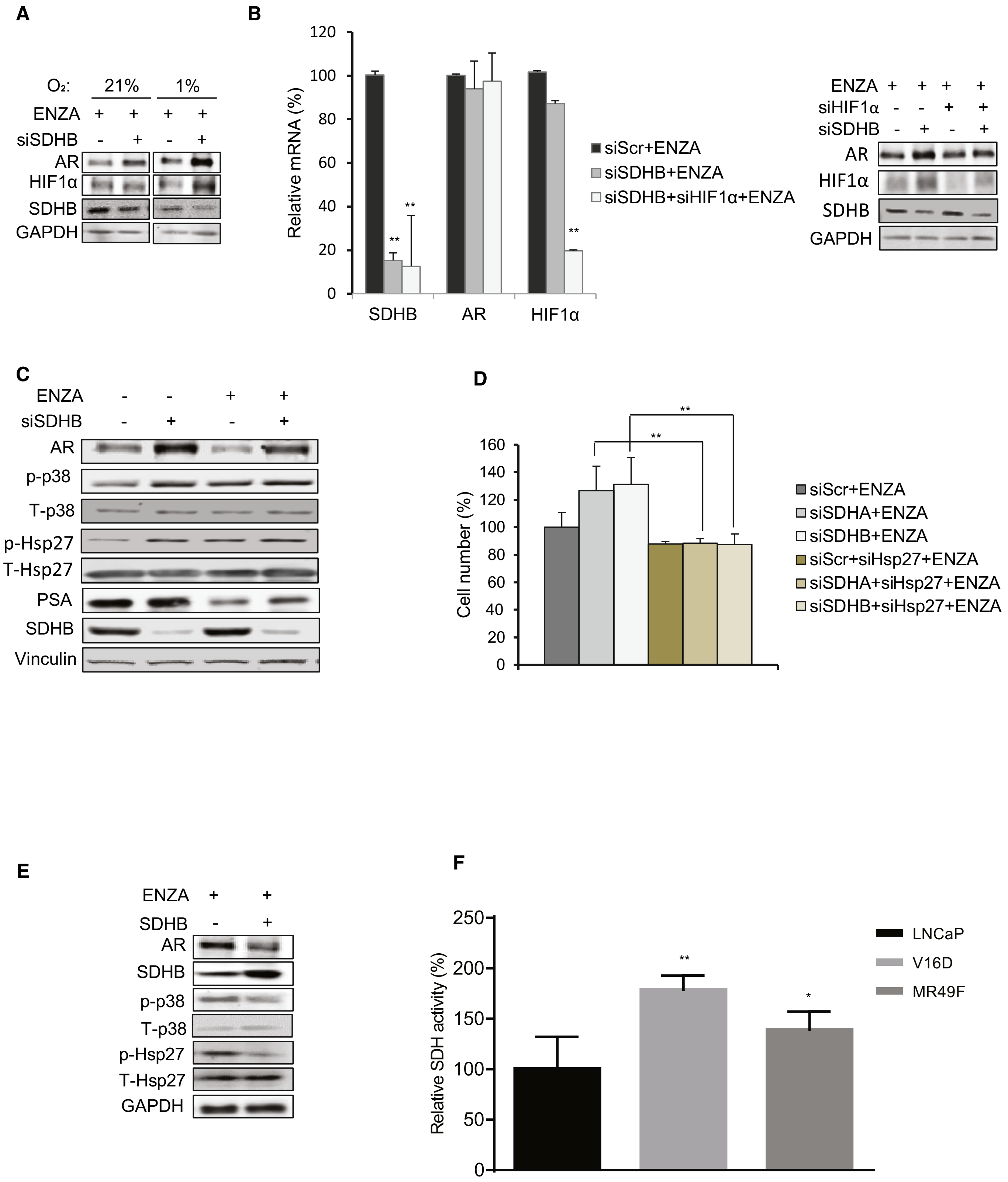
A
O₂: 21% 1%
ENZA + + + +
siSDHB - + - +
AR
HIF1α
SDHB
GAPDH
B
120
100
80
60
40
20
0
Relative mRNA (%)
SDHB AR HIF1α
**
**
**
siScr+ENZA
siSDHB+ENZA
siSDHB+siHIF1α+ENZA
ENZA + + + +
siHIF1α - - + +
siSDHB - + - +
AR
HIF1α
SDHB
GAPDH
C
ENZA - - + +
siSDHB - + - +
AR
p-p38
T-p38
p-Hsp27
T-Hsp27
PSA
SDHB
Vinculin
D
160
140
120
100
80
60
40
20
0
Cell number (%)
**
**
siScr+ENZA
siSDHA+ENZA
siSDHB+ENZA
siScr+siHsp27+ENZA
siSDHA+siHsp27+ENZA
siSDHB+siHsp27+ENZA
E
ENZA + +
SDHB - +
AR
SDHB
p-p38
T-p38
p-Hsp27
T-Hsp27
GAPDH
F
250
200
150
100
50
0
Relative SDH activity (%)
**
*
LNCaP
V16D
MR49F

Figure EV3.

Figure EV4. SDH repression activates p-CaMKK2/p-AMPK axis.

A SDHA/SDHB silencing in LNCaP cells increased levels of intracellular lactic acid as measured by LC-MS, while SDHA/SDHB overexpression decreased the levels compared with ENZA alone.

B SDHA or SDHB silencing increased ATP levels in LNCaP cells compared with ENZA alone.

C Treatment with AMPK inhibitor Dorsomorphin (10 μM, 24 h) inhibited AR-upregulation post siSDHB in LNCaP cells.

D AMPK silencing abrogated survival advantage imparted by SDH repression in LNCaP cells.

E, F Treatment of LNCaP cells with DMS (E) or ENZA (F) increased p-CaMKK2 and downstream p-AMPK/p-p38/p-Hsp27 axis and co-treatment with the CaMKK2 inhibitor STO-609 reduced AMPK phosphorylation and downstream signaling cascade induced by the two reagents.

Data information: ENZA: enzalutamide, DMS: Dimethyl succinate. Data shown as mean ± SD of three independent experiments. Statistical analysis was performed using one-way ANOVA followed by Tukey's test. *$P < 0.05$ and **$P < 0.01$ compared between groups. Exact *P* values are reported in the Appendix Table S7.

© 2021 The Authors

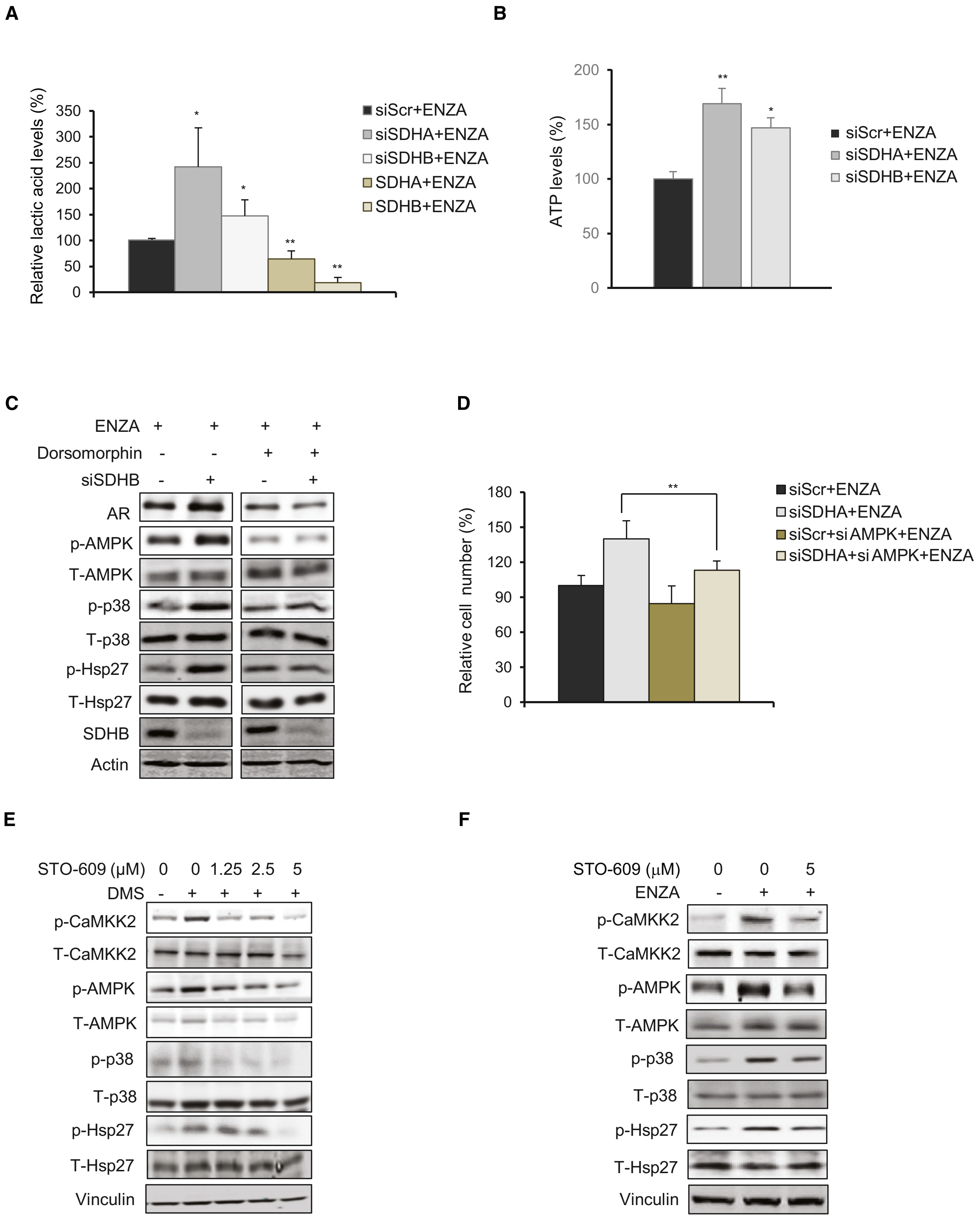
A
Relative lactic acid levels (%)
350
300
250
200
150
100
50
0
siScr+ENZA
siSDHA+ENZA
siSDHB+ENZA
SDHA+ENZA
SDHB+ENZA
B
ATP levels (%)
200
150
100
50
0
siScr+ENZA
siSDHA+ENZA
siSDHB+ENZA
C
ENZA + + + +
Dorsomorphin - - + +
siSDHB - + - +
AR
p-AMPK
T-AMPK
p-p38
T-p38
p-Hsp27
T-Hsp27
SDHB
Actin
D
Relative cell number (%)
180
150
120
90
60
30
0
siScr+ENZA
siSDHA+ENZA
siScr+si AMPK+ENZA
siSDHA+si AMPK+ENZA
E
STO-609 (μM) 0 0 1.25 2.5 5
DMS - + + + +
p-CaMKK2
T-CaMKK2
p-AMPK
T-AMPK
p-p38
T-p38
p-Hsp27
T-Hsp27
Vinculin
F
STO-609 (μM) 0 0 5
ENZA - + +
p-CaMKK2
T-CaMKK2
p-AMPK
T-AMPK
p-p38
T-p38
p-Hsp27
T-Hsp27
Vinculin

Figure EV4.

Figure EV5. TMA study on PCa patients and PDX tissues supports AR-SDH loop.

A Contingency plots give detailed picture of immunoexpression in terms of intensity levels for each marker across different stages in human PCa TMA. Untreated (n = 70), NHT (n = 130), and CRPC (n = 24).

B AR-low NEPC samples (n = 4) in human PCa TMA have reduced p-Hsp27 levels compared with AR-positive NEPC (n = 6).

C TMA analysis on LTL331 PDX tissues shows reduced SDHA and p-Hsp27 levels in AR-protein null LTL331R NEPC PDX compared with castration-sensitive adenocarcinoma LTL331.

D Relative immunoexpression of AR, SDHA, p-CaMKK2, p-AMPK, p-38, and p-Hsp27 in castration-sensitive (n = 14) and castration-resistant adeno-PC (n = 8) compared with NEPC tissues (n = 4) demonstrate similar trend of reduced SDHA and p-Hsp27 levels in AR-low NEPC PDX models compared to castration-sensitive or castration-resistant adeno-PC. Right panel table lists names of PDX models (see http://www.livingtumorlab.com/PDX_Prostate.html) used to build the TMA.

E, F GSEA analysis on AR positive CRPC vs AR negative NEPC samples enriched signature pathways (E) including androgen response in CRPCs and E2F signaling and G2/M checkpoint in NEPC tumors, respectively. SDHB-target pathways including oxidative phosphorylation, adipogenesis, and fatty acid metabolism enriched in AR-positive CRPCs whereas hypoxia and glycolysis were enriched in AR-negative NEPCs (F). Pathways enriched in CRPCs and NEPC samples are shown in red and purple colors, respectively.

Data information: All the TMAs were constructed from minimum 2 cores per patient. Data shown as means ± SEM by one-way ANOVA analysis followed by Tukey's test. *$P < 0.05$; **$P < 0.01$ and ****$P < 0.0001$ compared between groups. Exact P values are reported in the Appendix Table S7.

© 2021 The Authors

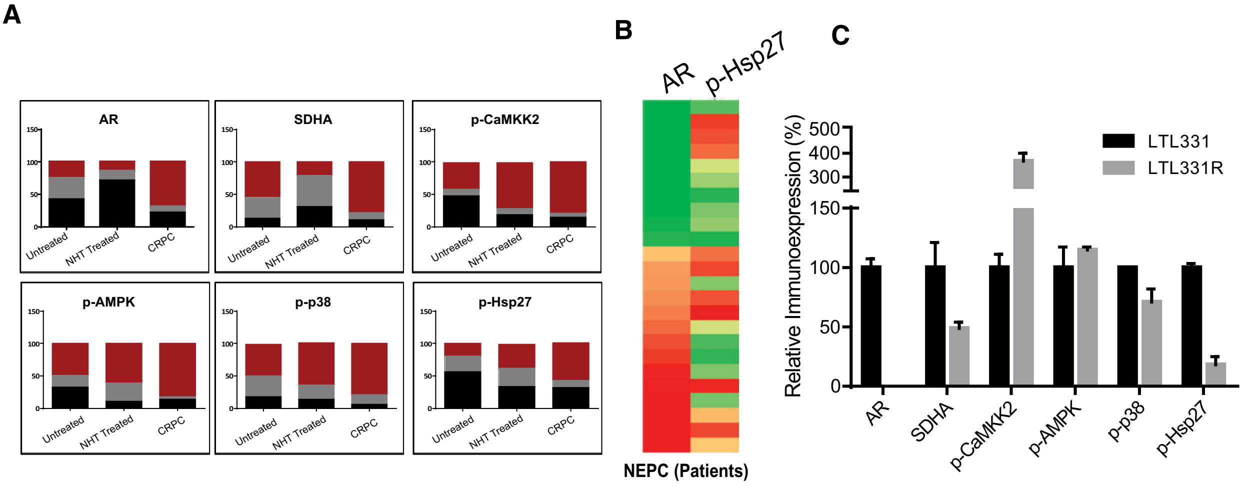

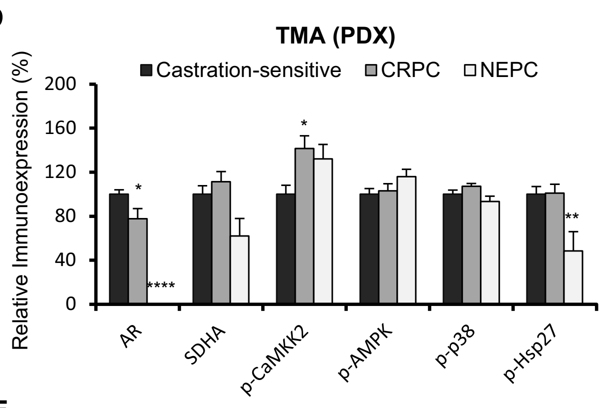

Castration sensitive	CRPC	NEPC
LTL310	LTL310R	LTL331
LTL311	LTL311R	LTL352
LTL313B	LTL313BR	LTL370
LTL313H	LTL313HR	LTL545
LTL418	LTL418R	
LTL467	LTL467R	
LTL484	LTL484R	
LTL471	LTL556	
LTL508		
LTL517		
LTL539		
LTL573		
LTL331		
LTL412		

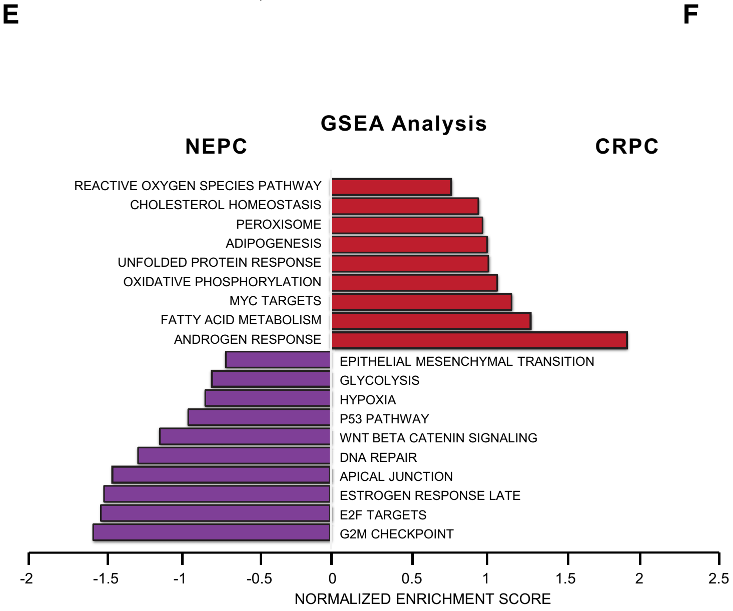

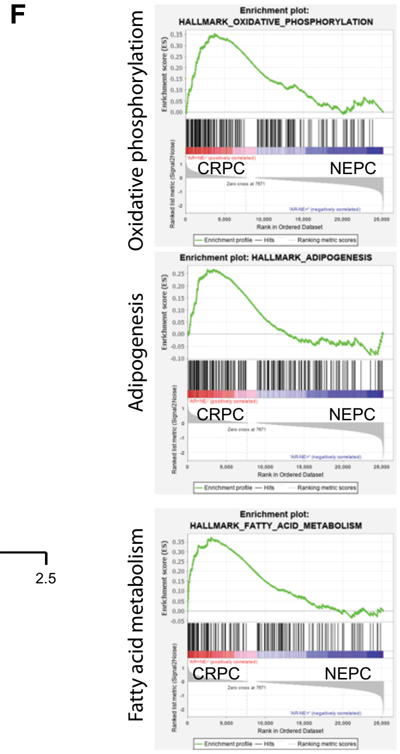

Figure EV5.
