# Supplementary figures and images for: Androgen receptor (AR) antagonism triggers acute succinate‐mediated adaptive responses to reactivate AR signaling
Source: EMBO Mol Med. 2021 Mar 11;13(5):e13427. doi: 10.15252/emmm.202013427 (PMC8103094; doi:10.15252/emmm.202013427)

Figure 2B

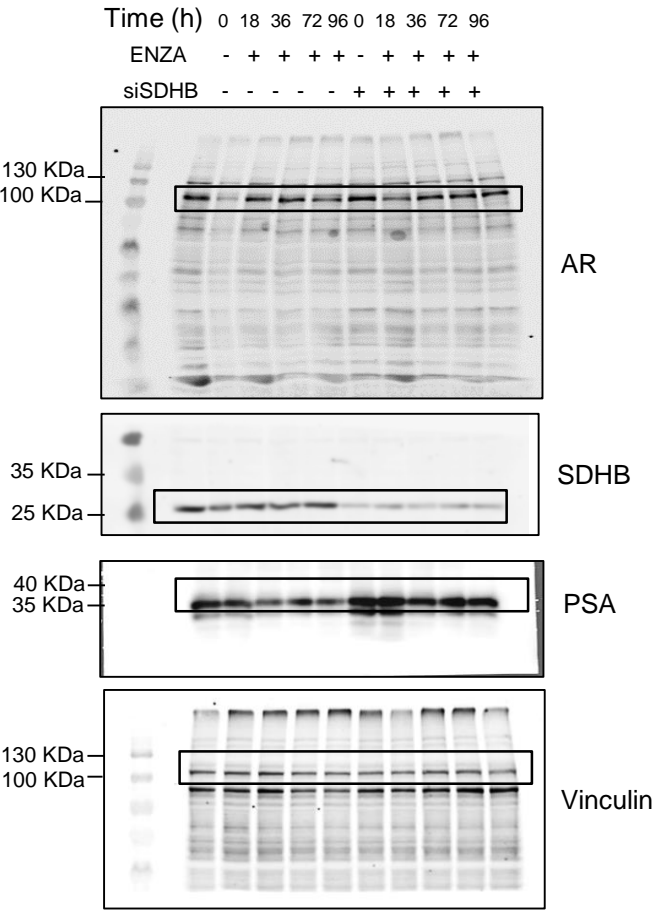

Supplement: Supplementary file 5 — Source Data for Figure 2 [file EMMM-13-e13427-s002.pdf]

Figure 3A

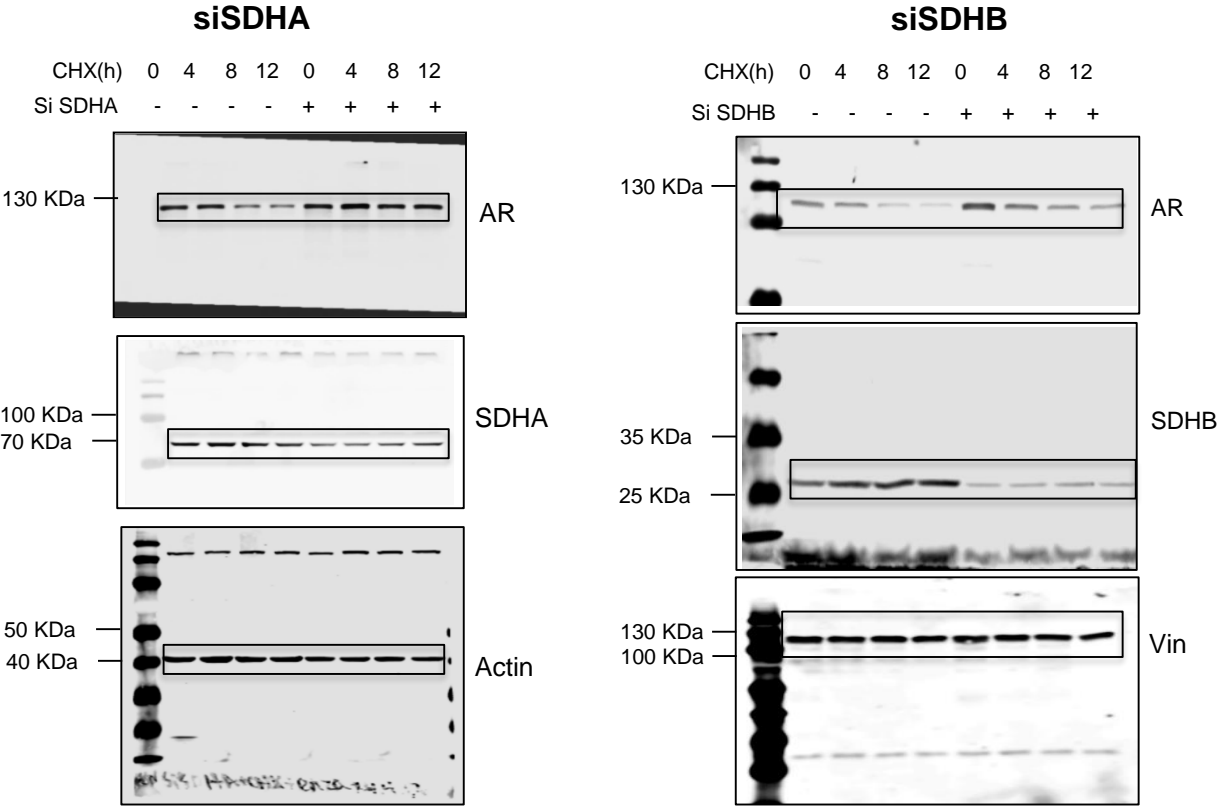

Supplement: Supplementary file 6 — Source Data for Figure 3 [file EMMM-13-e13427-s003.pdf]

Figure 4C

|        |   |   |   |   |   |   |
|--------|---|---|---|---|---|---|
| ENZA   | + | + | + | + | + | + |
| siScr  | - | + | - | - | + | - |
| siAMPK | - | - | - | + | + | + |
| siSDHA | + | - | - | + | - | - |
| siSDHB | - | - | + | - | - | + |

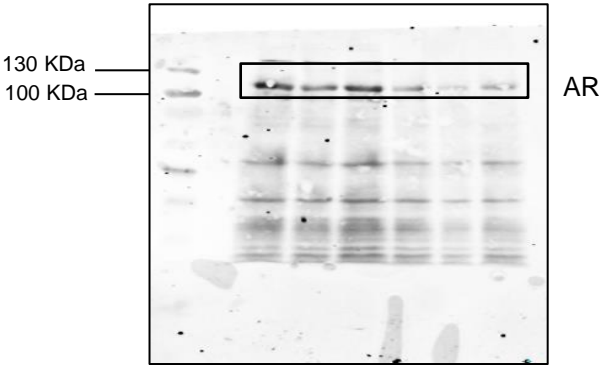

|        |   |   |   |   |   |   |
|--------|---|---|---|---|---|---|
| ENZA   | + | + | + | + | + | + |
| siScr  | - | + | - | - | + | - |
| siAMPK | - | - | - | + | + | + |
| siSDHA | + | - | - | + | - | - |
| siSDHB | - | - | + | - | - | + |

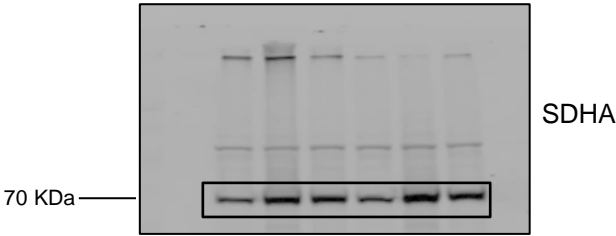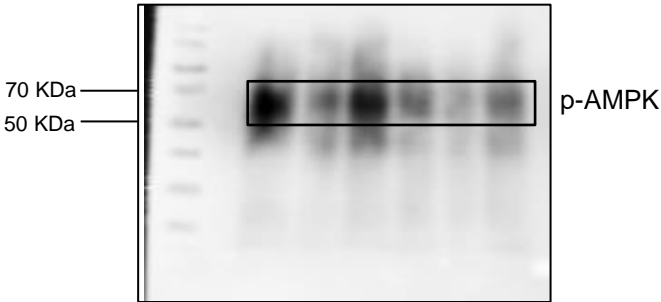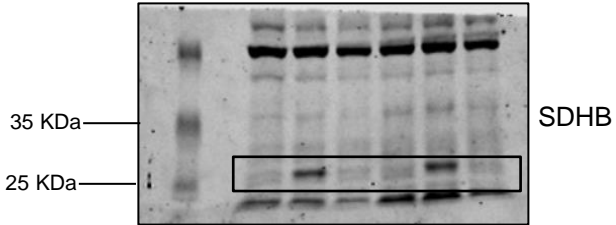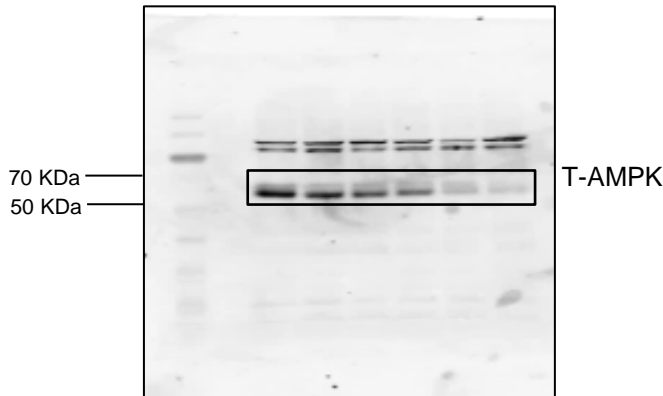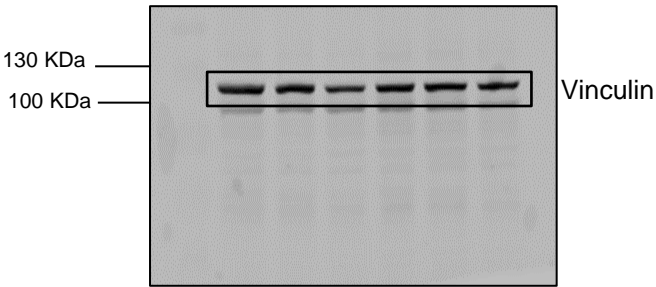

Supplement: Supplementary file 7 — Source Data for Figure 4 [file EMMM-13-e13427-s005.zip › Source_file_Figure_4C.pdf]

Figure 4E

|          |   |   |   |   |   |   |
|----------|---|---|---|---|---|---|
| ENZA     | + | + | + | + | + | + |
| siCaMKK2 | - | - | - | + | + | + |
| siScr    | - | + | - | - | + | - |
| siSDHA   | + | - | - | + | - | - |
| siSDHB   | - | - | + | - | - | + |

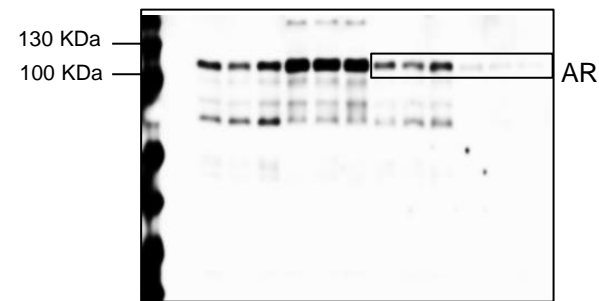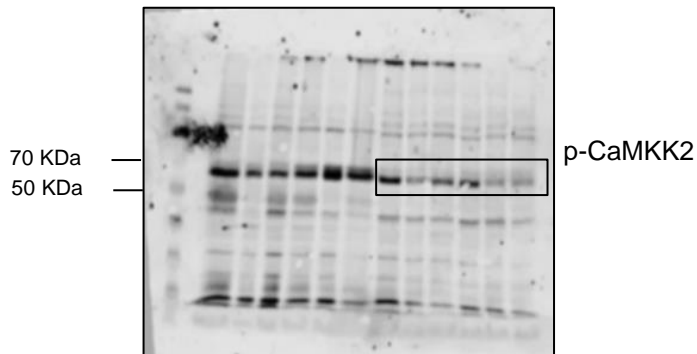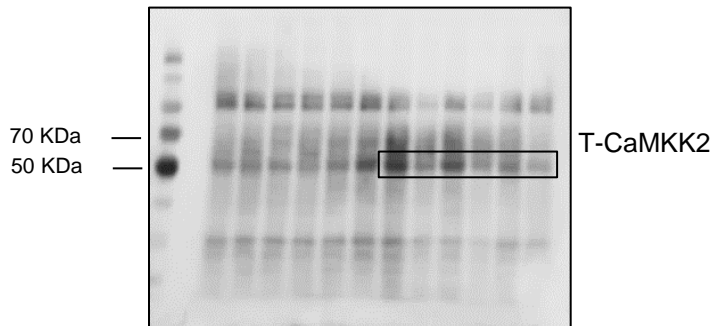

|          |   |   |   |   |   |   |
|----------|---|---|---|---|---|---|
| ENZA     | + | + | + | + | + | + |
| siCaMKK2 | - | - | - | + | + | + |
| siScr    | - | + | - | - | + | - |
| siSDHA   | + | - | - | + | - | - |
| siSDHB   | - | - | + | - | - | + |

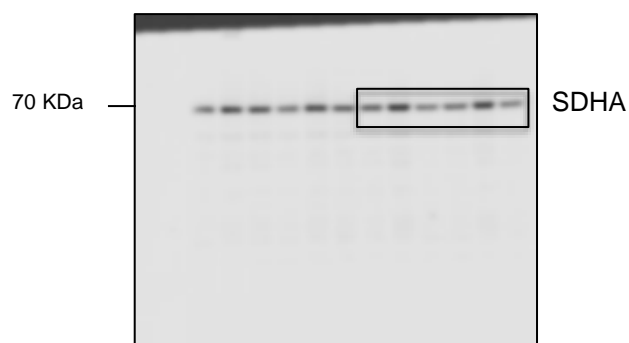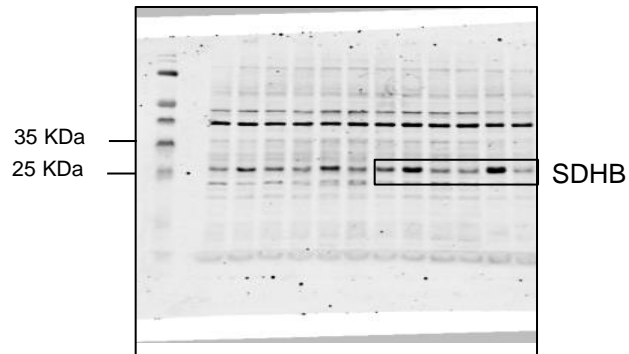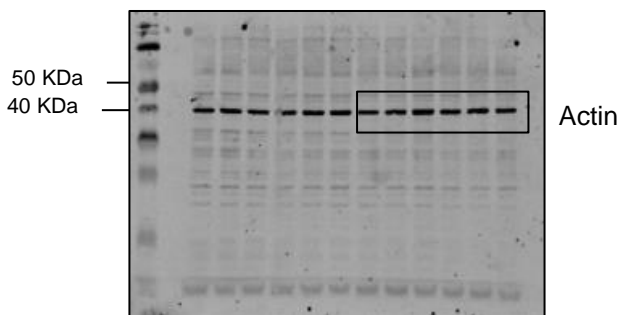

Supplement: Supplementary file 7 — Source Data for Figure 4 [file EMMM-13-e13427-s005.zip › Source_file_for_4E.pdf]

Figure 4D

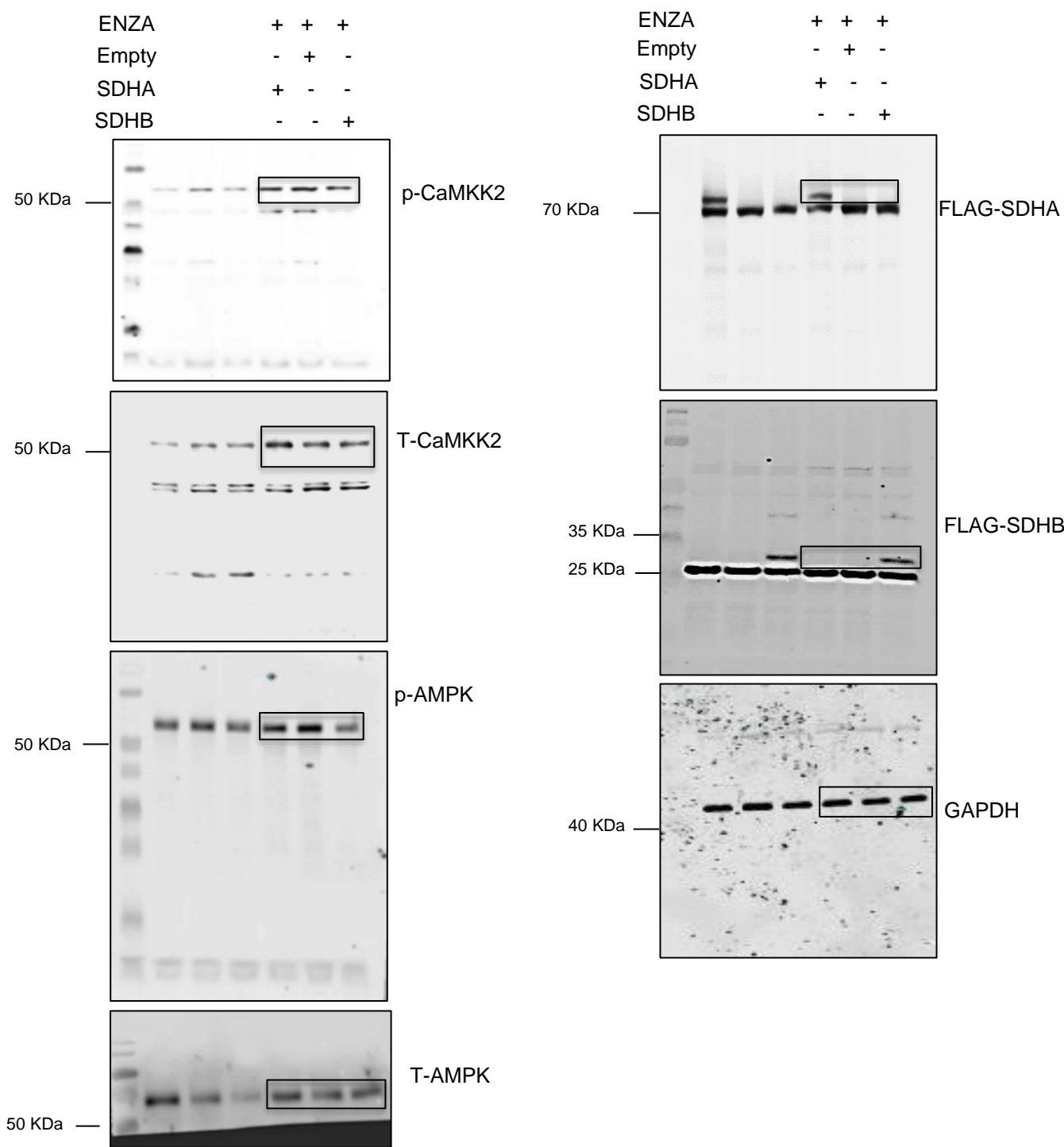

Supplement: Supplementary file 7 — Source Data for Figure 4 [file EMMM-13-e13427-s005.zip › Source_file_for_figure_4D.pdf]

Figure 4F

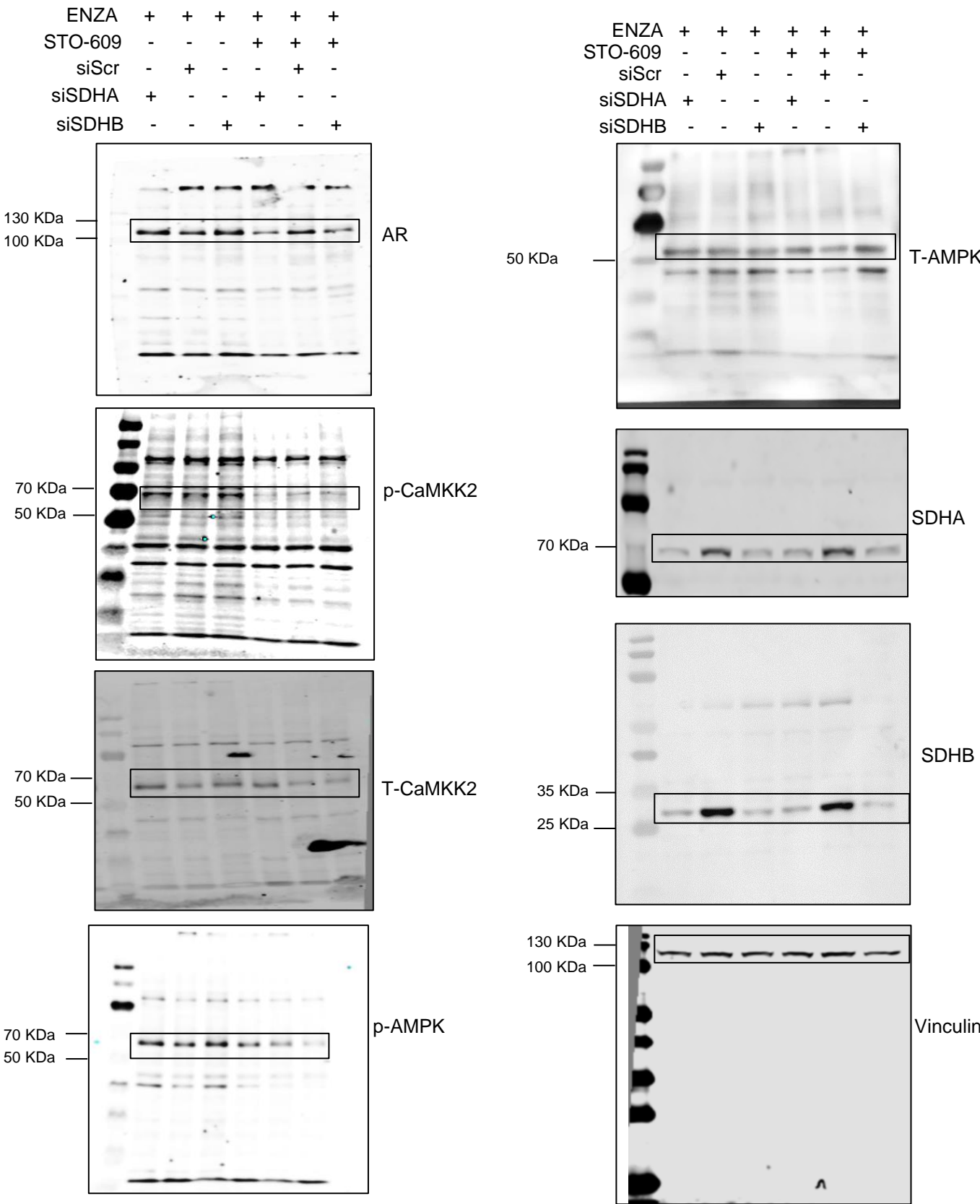

Supplement: Supplementary file 7 — Source Data for Figure 4 [file EMMM-13-e13427-s005.zip › Source_file_for_figure_4F.pdf]

Figure 6F

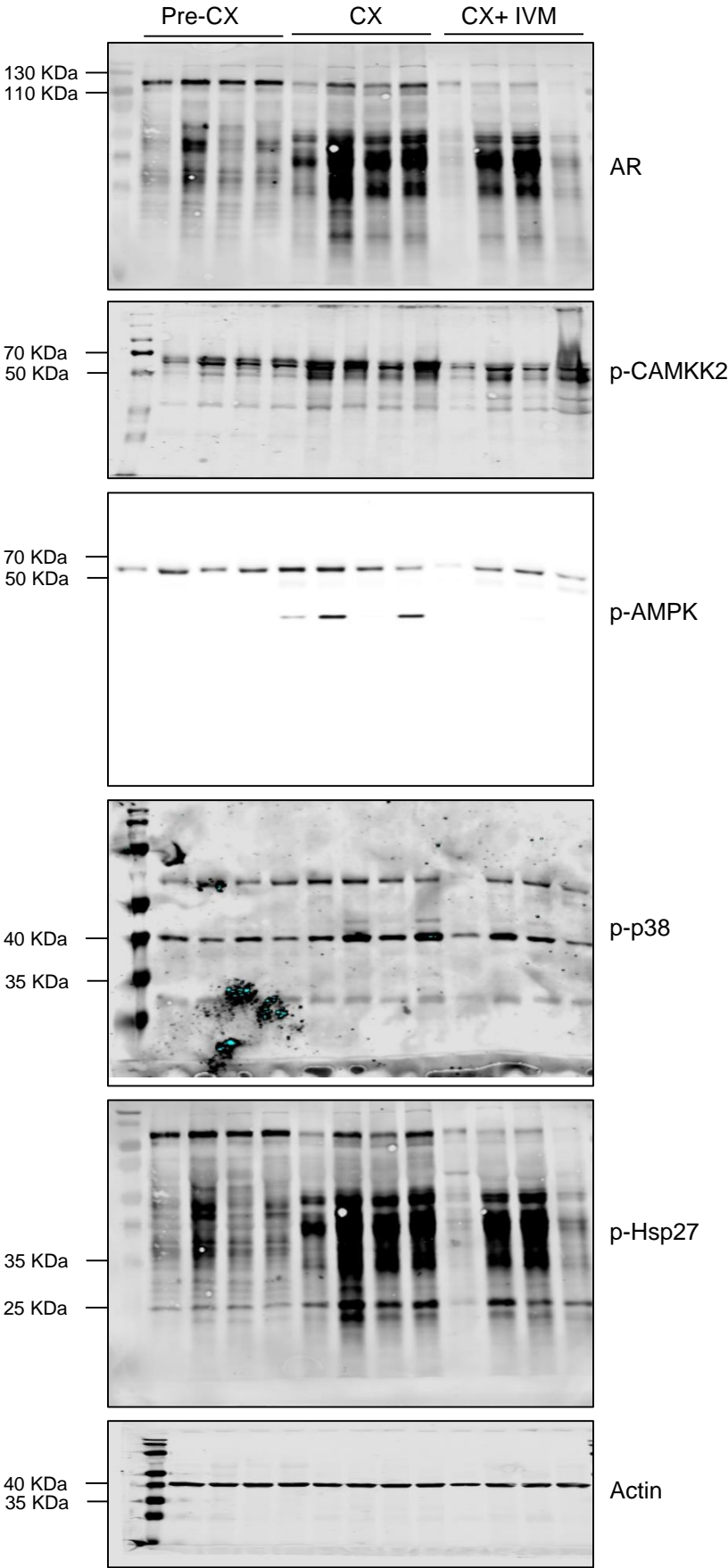

Supplement: Supplementary file 9 — Source Data for Figure 6 [file EMMM-13-e13427-s001.pdf]
